# Suspected Recurrent SARS-CoV-2 Infections Among Residents of a Skilled Nursing Facility During a Second COVID-19 Outbreak — Kentucky, July–November 2020

**DOI:** 10.15585/mmwr.mm7008a3

**Published:** 2021-02-26

**Authors:** Alyson M. Cavanaugh, Douglas Thoroughman, Holly Miranda, Kevin Spicer

**Affiliations:** ^1^Kentucky Department for Public Health; ^2^Epidemic Intelligence Service, CDC; ^3^Career Epidemiology Field Officer Program, CDC; ^4^Division of Performance Improvement and Field Services, Center for State, Tribal, Local and Territorial Support, CDC; ^5^Division of Healthcare Quality Promotion, National Center for Emerging and Zoonotic Infectious Diseases, CDC.

Reinfection with SARS-CoV-2, the virus that causes coronavirus disease 2019 (COVID-19), is believed to be rare (*1*). Some level of immunity after SARS-CoV-2 infection is expected; however, the evidence regarding duration and level of protection is still emerging (*2*). The Kentucky Department for Public Health (KDPH) and a local health department conducted an investigation at a skilled nursing facility (SNF) that experienced a second COVID-19 outbreak in October 2020, 3 months after a first outbreak in July. Five residents received positive SARS-CoV-2 reverse transcription–polymerase chain reaction (RT-PCR) test results during both outbreaks. During the first outbreak, three of the five patients were asymptomatic and two had mild symptoms that resolved before the second outbreak. Disease severity in the five residents during the second outbreak was worse than that during the first outbreak and included one death. Because test samples were not retained, phylogenetic strain comparison was not possible. However, interim period symptom resolution in the two symptomatic patients, at least four consecutive negative RT-PCR tests for all five patients before receiving a positive test result during the second outbreak, and the 3-month interval between the first and the second outbreaks, suggest the possibility that reinfection occurred. Maintaining physical distance, wearing face coverings or masks, and frequent hand hygiene are critical mitigation strategies necessary to prevent transmission of SARS-CoV-2 to SNF residents, a particularly vulnerable population at risk for poor COVID-19–associated outcomes.* Testing, containment strategies (isolation and quarantine), and vaccination of residents and health care personnel (HCP) are also essential components to protecting vulnerable residents. The findings of this study highlight the importance of maintaining public health mitigation and protection strategies that reduce transmission risk, even among persons with a history of COVID-19 infection.

## First Outbreak: Investigation and Findings

In July, a Kentucky SNF notified the local health department of a case of COVID-19 in one of the facility’s HCP; KDPH was also notified. RT-PCR testing was performed in accordance with state protocol to identify additional cases among residents and HCP. A confirmed COVID-19 case was defined as a positive RT-PCR test result for a SNF resident or HCP. The index patient in this outbreak was a symptomatic HCP. Initially, symptomatic persons and exposed residents who had received direct care and HCP who had close contact with the infected HCP were tested.^†^ Facility-wide testing for all residents and HCP began when additional positive test results were received. Residents and HCP who received negative results were retested weekly; in addition, anyone experiencing symptoms was tested at the time of symptom onset. Residents with positive test results were cohorted in a separate COVID-19 unit with dedicated HCP who used appropriate personal protective equipment. The SNF required the receipt of two negative test results collected >24 hours apart to release patients from the COVID-19 unit. HCP with positive test results could not return to work until completion of their isolation period.^§^ Residents who had been exposed to COVID-19 with negative test results were cohorted in a separate unit, primarily in double-occupancy rooms. Weekly testing of all noninfected HCP and residents continued for >14 days after the final case of the initial outbreak was identified. In total, 20 (17.4%) of 115 residents and five (3.5%) of 143 HCP in this facility received positive test results during July 16–August 11, representing an overall attack rate of 9.7%. Eight (40.0%) residents with COVID-19 were hospitalized, and five (25.0%) residents with COVID-19 died. No hospitalizations or deaths occurred among HCP with COVID-19.

KDPH and the local health department encouraged the facility to continue to monitor hand hygiene of residents and HCP, emphasize environmental cleaning and disinfection, practice universal masking, use standard precautions for general resident contact, quarantine newly-admitted and readmitted patients for 14 days, employ testing, and restrict visitation based on county-level incidence rates. The facility continued to monitor all residents and HCP for signs and symptoms of COVID-19 and to test symptomatic persons. The SNF continued to test HCP at least every other week between the two outbreaks. A total of 597 facility-ordered RT-PCR tests were performed in September, and 331 tests were performed during October 1–29; all results were negative.

## Second Outbreak: Investigation and Findings

On October 30, 2020, the same SNF notified the local health department and KDPH of two COVID-19 cases after two symptomatic residents received positive test results. Testing and cohorting practices similar to those implemented during the first outbreak were initiated, and testing of residents and HCP was increased to twice weekly. During October 30–December 7, a total of 85 (74.6%) of 114 residents and 43 (29.5%) of 146 HCP received positive SARS-CoV-2 RT-PCR test results, representing an attack rate of 49.2% among the 260 SNF residents and HCP present at the start of the outbreak in October. Among the 85 resident cases identified in the second outbreak, 15 (17.6%) patients died. No HCP died.

Among 12 residents who received positive test results during the first outbreak (July–August) and were still living in the facility in October, five also received positive results during the second outbreak >90 days after the date that their first specimens were collected. These patients were classified as having recurrent cases of COVID-19. Among the five HCP who had received a positive SARS-CoV-2 test result during the July outbreak, only one was working at the facility at the time of the second outbreak. This staff member did not have a positive SARS-CoV-2 test result during the second outbreak. KDPH performed SNF interviews, reviewed testing results from the National Electronic Disease Surveillance System, and contacted the testing laboratories to investigate exposures, testing history, and course of illness of the five patients identified as having recurrent COVID-19. The activity was reviewed by CDC and conducted consistent with applicable federal law and CDC policy.^¶^

The five patients with recurrent COVID-19 ranged in age from 67 to 99 years; four were women (Table). Each of the five patients had more than three chronic underlying health conditions, and all were permanent residents of the SNF. None of the patients with recurrent COVID-19 had an immunosuppressive condition or was taking immunosuppressive medications that might have hindered clearance of the virus or predisposed them to virus reactivation (*3*).

**TABLE:** Demographic and clinical characteristics and laboratory test results among five skilled nursing facility residents with recurrent COVID-19 **—** Kentucky, 2020

Patient	Sex (age group, yrs)	First outbreak (Jul–Aug)		Second outbreak (Oct–Dec)		
		Ct values*	Symptoms	No. of days since positive test result in first outbreak	Ct values*	Symptoms
A	M (80–89)	N1: 28.5	Asymptomatic	101	N1: 30.0	Functional decline, lethargy, decreased appetite, dry cough; onset 1 day before test, persisted 14 days
		N2: 29.0			N2: 31.0	
		RNAse P: 24.4			RNAse P: 32.0	
B	F (80–89)	N1: 28.2	Asymptomatic	103	N1: 17.5	Congestion, SOB, respiratory failure; onset and hospitalization 1 day after test, death 8 days later
		N2: 28.8			N2: 19.1	
		RNAse P: 25.8			RNAse P: 25.0	
				104^†^	E: 18.2	
					N: 19.8	
C	F (60–69)	N1: 28.9	Nausea at day 13 after positive test, persisted 1 day	109	N1: 19.3	Cough, SOB, sore throat, loss of appetite, malaise, muscle aches; onset day of test, persisted 17 days
		N2: 28.9			N2: 20.4	
		RNAse P: 24.9			RNAse P: 27.2	
D	F (70–79)	N1: 29.2	Gastrointestinal symptoms, onset 4 days prior to test, persisted 17 days, no fever or respiratory symptoms	109	N1: 18.5	Loss of appetite, malaise; onset 3 days after test, persisted 12 days
		N2: 29.6			N2: 18.9	
		RNAse P: 25.7			RNAse P: 22.2	
E	F (90–99)	N1: 28.9	Asymptomatic	110	N1: 17.2	Cough, loss of appetite, malaise, muscle aches; onset day of test, persisted 6 days
		N2: 29.9			N2: 17.9	
		RNAse P: 33.0			RNAse P: 21.1	

**Abbreviations:** COVID-19 = coronavirus disease 2019; Ct = cycle threshold; F = female; M = male; RT-PCR = reverse transcription–polymerase chain reaction; SOB = shortness of breath.

* E and N genes are gene targets used to detect infection with SARS-CoV-2, the virus that causes COVID-19. One or both of the N1 and N2 gene targets must be detected with a Ct value ≤37 for a positive result. Lower Ct values indicate higher concentrations of SARS-CoV-2. RNAse P is a control that is used to assess specimen quality. Ct values ≤37 indicate the presence of human RNAse P gene.

^†^ Patient B was retested with RT-PCR on hospital admission.

Among these five patients, only two (patients C and D) were symptomatic during the first outbreak; neither had fever or respiratory symptoms, and neither was hospitalized (Figure). Both had complete resolution of symptoms between the two outbreaks. All residents with recurrent COVID-19 had at least four consecutive negative RT-PCR test results between their two positive tests. All five patients received their positive RT-PCR results for the second COVID-19 diagnosis in the midst of the second facility outbreak and therefore after facility exposure to SARS-CoV-2. Three patients (patients A, C, and D) with recurrent infection had roommates who received positive SARS-CoV-2 RT-PCR results before they received their own positive test results, confirming direct exposure. Patient B was in a private room, and patient E had a roommate who did not have COVID-19. Although no direct route of exposure was identified for patients B or E, exposure to SARS-CoV-2 was very likely because of the large number of infected persons in the facility during the second outbreak. Cycle threshold (Ct) values ≤30 were reported for positive test results for the five patients in each infectious episode, which suggests at least moderate upper respiratory tract viral loads (*4*). Although three of the five patients with recurrent COVID-19 were asymptomatic during their first infectious episode, all five experienced symptoms during their second infectious episode; the two patients who were symptomatic during the first outbreak experienced more severe symptoms during the second infectious episode compared with the symptoms they had during the first outbreak (Table). One resident patient required hospitalization and subsequently died.

**FIGURE:**
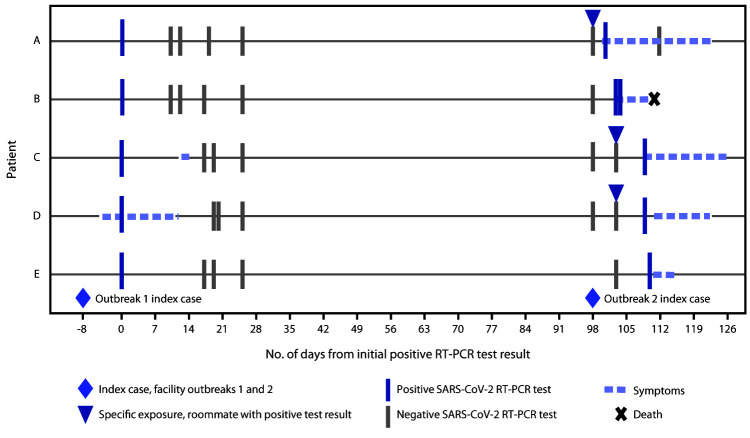
Exposure, symptom onset, and testing timeline for five patients with recurrent COVID-19 cases in a skilled nursing facility — Kentucky, July–December 2020* **Abbreviations:** COVID-19 = coronavirus disease 2019; RT-PCR = reverse transcription–polymerase chain reaction. * Dates indicate day of specimen collection.

## Discussion

After receiving positive COVID-19 test results during a SNF outbreak and subsequently receiving four to five negative SARS-CoV-2 RT-PCR test results, five residents received positive results >90 days later during the facility’s second COVID-19 outbreak, suggesting SARS-CoV-2 reinfection. All patients with recurrent COVID-19 experienced more severe disease during the second outbreak, and one died. The exposure history, including the timing of roommates’ infections and the new onset of symptoms during the second outbreak, suggest that the second positive RT-PCR results represented new infections after the patients apparently cleared the first infection.

The finding that all five patients with recurrent COVID-19 had either asymptomatic or mildly symptomatic courses during their first infections is noteworthy, suggesting the possibility that asymptomatic or mildly symptomatic initial infections do not produce a sufficiently robust immune response to prevent reinfection (*5*). The patients with recurrent illness ranged in age from 67 to 99 years; a decline in immune system function with aging is well-documented, but little scientific evidence is available to date regarding whether or how an aging immune system might affect response to initial SARS-CoV-2 infection, likelihood of reinfection upon new exposure, and illness severity associated with reinfection (*6*). As with any diagnostic test, false-positive results are possible. The absence of symptoms in three of five patients during the initial episode could support the argument that the test results during the first outbreak were false positives, although it is known that up to 40%–50% of infections are asymptomatic (*7*,*8*). The probability that all five tests were false positives is a less likely explanation, especially in the context of a facility outbreak with associated severe morbidity and mortality. In addition, Ct values for the positive test results in the first outbreak were within the cutoff for limit of detection, suggesting virus titers consistent with infection.

These findings highlight the importance of maintaining public health practices that reduce transmission risk, even among persons who have previously received a positive SARS-CoV-2 test result. These findings support the possibility of reinfection in this population, though more definitive evidence with genomic sequencing is missing. The findings also suggest the possibility that disease can be more severe during a second infection.

The findings in this report are subject to at least three limitations. First, because specimens were not stored, genomic sequencing to confirm a reinfection was not possible (*9*). Second, no additional testing was performed during the first outbreak until at least 10 days after the first RT-PCR positive test result for the five residents later identified to have recurrent COVID-19. Therefore, no additional test results exist to support the initial test result as a true positive. Finally, no serologic testing was performed after the first outbreak, which could have helped confirm infection before the second infectious episode.

Five SNF residents received positive SARS-CoV-2 test results during two separate facility outbreaks that occurred in July and October 2020, suggesting possible reinfection. Affected persons experienced more severe illness during their second SARS-CoV-2 infection. Reinfection risk to the general population is suspected to be low, but SNF residents might have higher risk for new exposures, given the congregate nature of these settings and ongoing interactions with HCP and other residents. In addition, the level and duration of postinfection immunity in persons with an aging immune system is unknown, but the potential health consequences of reinfection among SNF populations remain serious. Therefore, steps to protect this population from the ongoing potential of SARS-CoV-2 exposures should be implemented. Based on the observations of this study, testing and cohorting practices in SNFs should not assume that residents infected >90 days earlier are immune to COVID-19. Public health interventions to limit transmission are vital for all persons in SNFs, including those who have previously been infected with SARS-CoV-2; these include physical distancing, use of masks (including by SNF residents, if tolerated), and frequent hand hygiene using hand sanitizer with 60%–95% alcohol or washing with soap and water for at least 20 seconds. Vaccination in these settings, as recommended by the Advisory Committee on Immunization Practices, is particularly important to optimally protect these vulnerable persons (*10*).

**Summary**

**What is already known about this topic?**

Case reports of reinfection with SARS-CoV-2 exist; however, data are limited as to the frequency and outcomes of reinfection.

**What is added by this report?**

Five residents of a skilled nursing facility received positive SARS-CoV-2 nucleic acid test results in two separate COVID-19 outbreaks separated by 3 months. Residents received at least four negative test results between the two outbreaks, suggesting the possibility of reinfection. Severity of disease in the five residents during the second outbreak was worse than that during the first outbreak and included one death.

**What are the implications for public health practice?**

Skilled nursing facilities should use strategies to reduce the risk for SARS-CoV-2 transmission among all residents, including among those who have previously had a COVID-19 diagnosis. Vaccination of residents and health care personnel in this setting is particularly important to protect residents.

## Footnotes

*
https://www.cdc.gov/coronavirus/2019-ncov/hcp/long-term-care.html

†
https://www.cdc.gov/coronavirus/2019-ncov/hcp/nursing-homes-responding.html

§ At the time of the first outbreak, KDPH guidance for return to work for HCP recommended a time- and symptom-based approach. https://chfs.ky.gov/agencies/dph/covid19/Guidanceforreleasefromisolation.pdf

¶ 45 C.F.R. part 46.102(l)(2).
